# Dialysis Service in the Embattled Tigray Region of Ethiopia: A Call to Action

**DOI:** 10.1155/2022/8141548

**Published:** 2022-06-24

**Authors:** Ephrem Berhe, Will Ross, Hale Teka, Hiluf Ebuy Abraha, Lewis Wall

**Affiliations:** ^1^College of Health Sciences, Ayder Comprehensive Specialized Hospital, Mekelle University, Mek'ele, Ethiopia; ^2^Washington University School of Medicine, St. Louis, Missouri, USA

## Abstract

Haemodialysis is extremely limited in low-income countries. Access to haemodialysis is further curtailed in areas of active conflict and political instability. Haemodialysis in the Tigray region of Ethiopia has been dramatically affected by the ongoing civil war. Rapid assessment from the data available at Ayder Hospital's haemodialysis unit registry, 2015–2021, shows that enrollment of patients in the haemodialysis service has plummeted since the war broke out. Patient flow has decreased by 37.3% from the previous yearly average. This is in contrary to the assumption that enrollment would increase because patients could not travel to haemodialysis services in the rest of the country due to the complete blockade. Compared to the prewar period, the mortality rate has doubled in the first year after the war broke out, i.e., 28 deaths out of 110 haemodialysis recipients in 2020 vs. 43 deaths out of 81 haemodialysis recipients in the year 2021. These untoward outcomes reflect the persistent interruption of haemodialysis supplies, lack of transportation to the hospital, lack of financial resources, and the unavailability of basic medications due to the war and the ongoing economic and humanitarian blockade of Tigray in Northern Ethiopia. In the setting of this medical catastrophe, the international community should mobilize to advocate for resumption of life-saving haemodialysis treatment in Ethiopia's Tigray region and put pressure on the Ethiopian government to allow the passage of life-saving medicines, essential medical equipment, and consumables for haemodialysis into Tigray.

## 1. Introduction

**Figure 1:**
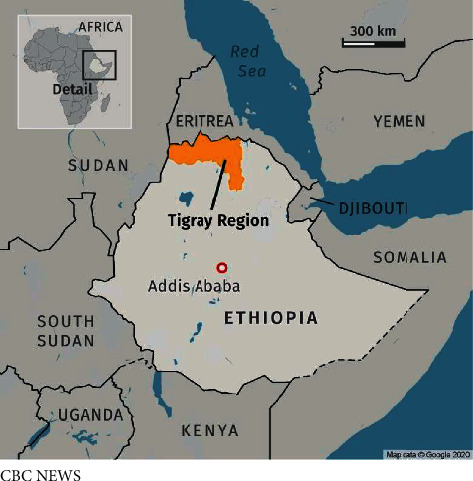
The area of conflict in Tigray region, Northern Ethiopia. Google Maps 2020.

The war in between Ethiopia's federal government and Tigray's regional government (Figure 1) broke out on November 4, 2020, and is still ongoing [1]. Twenty months on, displacement, death, and destruction have become the defining elements of this war, putting the lives of millions of civilians at stake. More significantly, this armed conflict has inflicted enormous damage to Tigray region's healthcare system, rendering 70–80% of health facilities in the region dysfunctional [2]. This situation has worsened over the past 10 months due to the blockade, leaving millions of people without access to basic healthcare services. The collapse of the health sector in Tigray, compounded by the complete blockade of aid and medical supplies, banking service interruption, and transportation and communications blackouts, has led to a catastrophic humanitarian crisis [3, 4]. Ayder Hospital, the flagship health care institution in the region, is in a dire situation as essential supplies have plummeted.

Ayder Hospital enjoyed unparalleled progress and expansion of services in the last decade. When starting functioning 14 years back, this 500-bed hospital had humble beginnings. At the beginning, it struggled with severe shortages of health professionals, medical instruments, and financial constraints. In a short stride, however, this once empty hospital grew fast through the hard work and persistence of its staff, the unwavering support of the university management, and national and international partners.

Ayder Hospital's haemodialysis centre was established as a public-private-partnership model program, suitable for the low resource settings in treating both acute and chronic kidney problems [5]. It is equipped with 12 modern B- Braun Dialog plus haemodialysis machine and is the only dialysis centre in the catchment area for 9 million people from the Tigray region and the neighbouring districts in the Amhara and Afar regions. Patients with acute kidney injury (AKI) requiring haemodialysis have mainly been supported through charitable donations while patients with chronic kidney disease have been self-funded.

A hospital that was barely better than a countryside health post in the early days of its establishment grew to be a specialized referral centre a decade later serving a catchment area of 9 million people from Tigray, neighbouring districts of the Afar and Amhara regions. Before the war broke out, Ayder Hospital's annual patient visit has risen to nearly 300,000 patients and thousands of major surgeries, deliveries, and a wide variety of interventions and specialized treatments were being given. This article seeks to showcase the impact of war on the haemodialysis services in Ayder Hospital, in the embattled Tigray region of Ethiopia.

## 2. Methodology

All data were extracted from the Ayder Hospital's haemodialysis centre registry for the period from 2013 to 2021. Record review was conducted in compliance with the hospital's policy on patient care and safety. Count data are presented in the manuscript. Event data represented death of patients, and censored data included renal recovery, transferred, transplanted, and those who were still on dialysis. A two-sample *t*-test was used to compare the mortality rate before and during the war and the loss-to-follow-up data.

## 3. Results

From November 2020 to April 2022, 69 new patients had been enrolled to the haemodialysis centre of Ayder Hospital. Close to two-third of these patients (60.9%) suffered from CKD. From the data available between 2013 and 2021, 550 patients received haemodialysis, of whom 181(32.7%) suffered from AKI and 319 (58%) suffered from chronic kidney disease (CKD). In 2021, during the war period, there were a total of 81 patients who underwent dialysis services; 69 (82.5%) of whom were newly enrolled patients in the same year. All of these patients have started dialysis on an emergency basis. Nearly half of the patients who are newly enrolled to the haemodialysis centre had only one haemodialysis session per week (Table 1).

**Figure 2:**
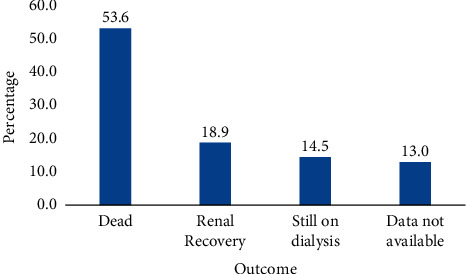
Outcomes of patients enrolled to the haemodialysis centre of Ayder Hospital in the year 2021.

Among the total patients who were under haemodialysis, more than half of the patients have progressively succumbed to death following interrupted haemodialysis sessions leading to suboptimal haemodialysis (Figure 2).

**Figure 3:**
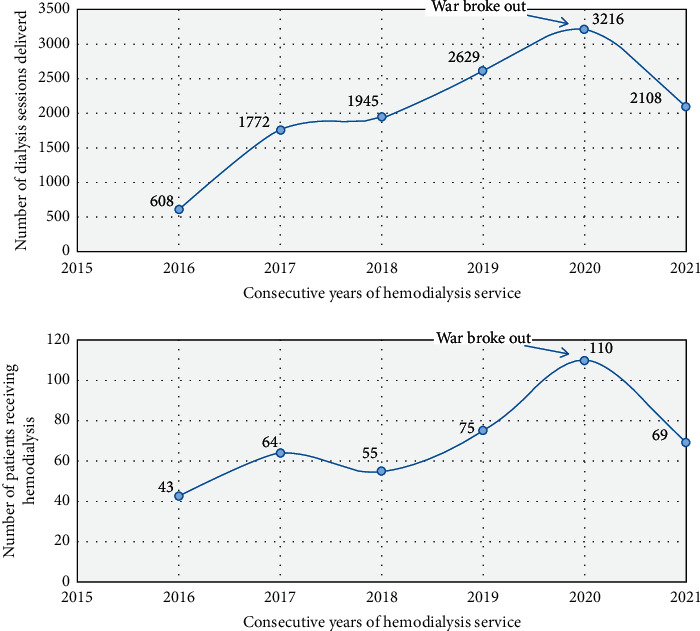
Data from Ayder Hospital haemodialysis centre registry displaying number of haemodialysis sessions delivered (upper) and number of patients enrolled to haemodialysis centre (lower) in each consecutive year from 2016 to 2021.

Hemodialysis utilization had been increasing annually before the war (Figure 3). The enrollment of patients in the haemodialysis service has decreased by 37.3% since the war broke out, in contrast to an annual increase by more than 30%. The impact of the war was immediately palpable such that both haemodialysis sessions and patient enrollment nose-dived to levels in 2019 (Figure 3). Mortality of patients under haemodialysis increased from 25.5% (28 deaths from 110 patients) in 2020 to 53% (43 deaths out of 81 patients) in 2021 (*p* < 0.05). Similarly, patient's lost-to-follow-up increased from 9 to 14% (11/81 vs. 10/110) but was not statistically significant (*p*=0.49). The registry from the centre shows between July 1, 2021, and January 15, 2022, 61 new patients (36 AKI, including 6 patients with pregnancy-related complications and 25 patients with CKD who needed emergency dialysis, succumbed owing to lack of haemodialysis supplies).

## 4. Discussion

The present study examines the sociodemographic, clinical characteristics, and outcomes of patients receiving haemodialysis services during an active war in the Tigray region of Northern Ethiopia. Similar to other studies, uremic features and pulmonary edema were the main reasons for haemodialysis initiation [6]. Almost half of the patients (47.8%) had only once weekly haemodialysis session. As the hospital is rendered incapable as a result of the complete blockade, we were not able to fully workup to reach at the specific cause of CKD in a third of the patients. Among those in whom we were able to determine the cause, diabetes and hypertension were the leading causes of kidney failure which is consistent with the findings of other studies in developed and developing countries [6, 7]. The emergent nature of haemodialysis offered to these patients was reflected such that 80% of the patients had a central catheter inserted regardless of the type of kidney failure (acute vs chronic).

Ethiopia's lack of available haemodialysis is unfortunately not unique among low-income countries. In a recent survey of haemodialysis availability in 126 countries [8], the median global use of haemodialysis was 298.4 per million populations (pmp). Access was considerably lower in low-income countries: 5.8 pmp in Ethiopia, 2.8 pmp in Zimbabwe, and 0.5 pmp in Tanzania. In addition, patients in low-income countries such as Ethiopia had to pay 100% of haemodialysis costs out of pocket. Delivering renal replacement therapy for end stage kidney disease (ESKD) in man-made conflict settings poses added challenges beyond what is required in resource-limited situations as it relies on factors that may be easily disrupted during times of armed conflict [9].

Now, in the background of destruction caused by the war, the haemodialysis centre at the Ayder Hospital has had to cut its functioning due to dwindling supplies, leaving twenty-two ESKD patients who had been dialysed for years to die.

Since July 2021, the haemodialysis center has been particularly affected as supplies that normally would have been brought north through the capital of Addis Ababa are no longer permitted to reach Ayder Hospital. The care provision in the embattled Tigray region is uniquely unprecedented, as patients who have ESKD could neither be offered optimal dialysis nor can they be referred to the transplant center in the capital Addis Ababa or abroad. As a result, hospital staff is continually forced to make tough decisions and to employ improvised, nonstandard management protocols. To meet the minimum needs of patients from across Tigray, the haemodialysis center had to reduce the number of haemodialysis sessions from twice weekly to once weekly or even as low as only once every fortnight. Lack of optimum haemodialysis sessions increases patients' suffering and leads to higher mortality, especially when patients have to settle for only one haemodialysis session per week or even one session every two weeks, whereas the maintenance of haemodialysis is typically prescribed thrice weekly [10, 11].

When the blockade tightened, the haemodialysis centre was forced to reuse single-use dialyzers for 6 to 8 sessions (or until the patient died). The staff are also being forced to use expired central venous catheters to gain vascular access and even resorted to desperate life-saving measures such as exchanging dialyzers among patients who died. Advising haemodialysis patients to stop their life-sustaining treatment because there are no longer sufficient medical resources, or haemodialysis supplies has become the daily norm for the haemodialysis team in Tigray.

**Figure 4:**
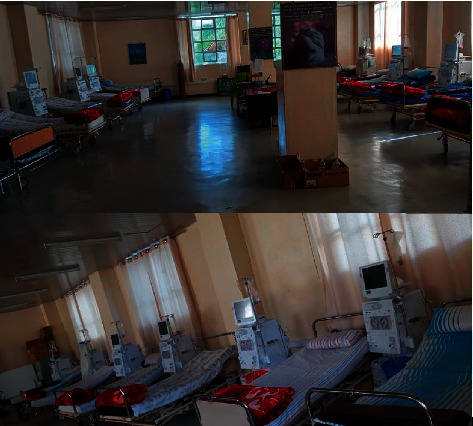
A picture of Ayder Hospital's haemodialysis centre displaying empty beds and having no patients connected to the haemodialysis machine due to lack of haemodialysis consumables.

Currently, Ayder Hospital cannot sustain even these markedly compromised and suboptimal haemodialysis services. The 25 remaining chronic haemodialysis patients, who have had their haemodialysis interrupted for the reasons stated above, are desperately waiting for haemodialysis consumables to arrive (Figure 4). The lack of resources at the haemodialysis centre is just one of the many problems encountered on a daily basis at Ayder Hospital [12].

The staggering mortality numbers during the war reflect the persistent interruption of haemodialysis supplies, lack of transportation to the hospital, lack of financial resources, and the unavailability of basic medications due to the war and the ongoing economic and humanitarian blockade of Tigray [13, 14]. Kidney failure patients in Tigray are struggling to obtain regular haemodialysis sessions amid the war, and as a result, all have ended up receiving suboptimal haemodialysis. Patients who could have been saved easily with haemodialysis are now dying due to this suboptimal care.

In addition to these adult patients, the hospital has been providing paediatric haemodialysis services for 10–12 patients per year. The youngest such patient was a 6-year-old child. These patients had an average of six sessions of haemodialysis for acute kidney failure. Over the past 10 months, only one paediatric patient has received haemodialysis, and five paediatric patients with AKI who could have been saved under normal circumstances have died due to lack of available haemodialysis supplies and central venous catheters.

Since July 2021, the dialysis team has carried out improvised peritoneal dialysis using locally prepared fluids using available instruments and intravenous fluids. Three patients received such treatment, and two patients survived. The haemodialysis team is suffering from enormous psychological stress as a result of witnessing the painful deaths of patients for whom they have cared for years, as well as the probable adverse outcomes that will befall the new patients needing haemodialysis.

## 5. Conclusion

Hundreds of patients with kidney failure who are in dire need of haemodialysis services in the Tigray region of Ethiopia are caught in war. As the blockade imposed on Tigray tightens, patients are left with no access to basic life-saving care including haemodialysis services. With no haemodialysis supplies reaching Ayder Hospital, patients with kidney failure are sent home to die. Few months ago, the hospital made an appeal to the world, asking the Ethiopian government to lift the blockade and the broader international community and nongovernmental organizations for support [15]. Without such support, the clock is running out for these patients, who will succumb to a slow, bitter, painful but avoidable death in front of the eyes of the attending medical staff. The international organizations with the mandate to protect civilians must act decisively to end the suffering of civilians who have no agency in the war. As nothing remains at the disposal of the staff caring for patients such as haemodialysis recipients, the international community and organizations with the capacity to help must organize funds to support Ayder Hospital. The Ethiopian government must allow the passage of life-saving medicines, essential medical equipment, and consumables for haemodialysis into Tigray. We demand that the relevant bodies including the United Nations, African Union, World Health Organization, Nephrology organizations, and, above all, the Ethiopian federal government (which has the primary responsibility) stand by the health care workers and the patients of Tigray.

## Figures and Tables

**Table 1:** Sociodemographic and clinical characteristics of the study participants, Ayder Comprehensive Specialized Hospital, Mekelle, Northern Ethiopia, 2021 (*n* = 69).

Characteristics	All cases *n* = 69	Outcomes	
		Event *n* = 37	Censored *n* = 32
Age in years (mean (SD))	40.5 (19.1)	47.4 (18.0)	32.5 (17.5)
Sex, *n* (%)			
Male	39 (56.5)	22 (56.4)	17 (43.6)
Female	30 (43.5)	15 (50.0)	15 (50.0)
Address, *n* (%)			
Tigray	65 (94.2)	34 (52.3)	31 (47.7)
Others	4 (5.8)	3 (66.7)	1 (33.3)
Type of kidney disease, *n* (%)			
AKI	27 (39.1)	15 (55.6)	12 (44.4)
CKD	42 (60.9)	22 (52.4)	20 (47.6)
LOS in days (median (IQR))	32 (83)	32 (55)	30.5 (117.5)
Number of dialysis indications, *n* (%)			
One indication	25 (36.2)	13 (52.0)	12 (48.0)
Two indications	37 (53.6)	18 (48.7)	19 (51.3)
Three indications	7 (10.2)	6 (85.7)	1 (14.3)
Indication, *n* (%)			
Uremic features	51 (73.9)	26 (51)	25 (49)
Pulmonary edema	28 (50.6)	14 (50.0)	14 (50.0)
Metabolic acidosis	27 (39.1)	21 (77.8)	6 (22.2)
Refractory hyperkalemia	14 (20.3)	6 (42.9)	8 (57.1)
Dialysis frequency (per week), *n* (%)			
Only once	33 (47.8)	13 (39.4)	20 (60.6)
Twice	36 (52.2)	24 (66.7)	12 (33.3)
Vascular access, *n* (%)			
Right IJV catheter	57 (82.6)	34 (59.7)	23 (40.3)
Arteriovenous fistula	9 (13.0)	2 (22.2)	7 (77.8)
Permanent central catheter	3 (4.4)	1 (33.3)	2 (67.7)
Causes of kidney failure, *n* (%)			
CKD of unknown cause	23 (33.3)	12 (52.2)	11 (47.8)
Diabetes	16 (23.2)	11 (68.7)	5 (31.3)
Hypertension	8 (11.6)	4 (50.0)	4 (50.0)
Sepsis	7 (10.1)	4 (57.1)	3 (42.8)
RPGN	4 (5.9)	0 (0.00)	4 (100.0)
Obstructive uropathy	3 (4.4)	2 (66.7)	1 (33.3)
Others	8 (11.7)	4 (50.0)	4 (50.0)

Event represents death of patients and censored includes cured, defaulted, transferred, transplanted, and those who are still on dialysis. AKI: acute kidney injury, CKD: chronic kidney disease, HIV: human immune deficiency virus, IJVC: internal jugular vein catheter. SD: standard deviations, LOS: length of stay. Uremic features include symptoms such as persistent nausea/vomiting, fatigue, anorexia, mental status changes, pruritus, and pericarditis. Other causes of kidney failure include drug nephrotoxicity (*n* = 2), HIV infection (*n* = 2), hypertensive disorders of pregnancy (*n* = 2), prerenal AKI, and malaria (*n* = 1 each), and others in address represent 1 Afar region and 3 Amhara regions.

## Data Availability

All relevant data are within the manuscript.
